# Zinner syndrome incidentally diagnosed in a man with ureteropelvic junction stone and hydronephrosis: A case report

**DOI:** 10.1016/j.eucr.2025.102930

**Published:** 2025-01-03

**Authors:** Tzu-Yu Chiu, Szu-Ju Chen, Chun-Lin Huang, Chi-Ping Huang, Wen-Chi Chen

**Affiliations:** a Department of Medical Education, China Medical University Hospital, Taiwan; b Department of Urology, Taichung Veterans General Hospital, Taichung, Taiwan; c Department of Radiology, China Medical University Hospital, Taiwan; d Department of Urology, China Medical University Hospital, Taiwan; e School of Medicine, School of Chinese Medicine, China Medical University, Taiwan; f Graduate Institute of Integrated Medicine, School of Chinese Medicine, China Medical University, Taiwan

**Keywords:** Zinner syndrome, Polysplenia syndrome, Renal agenesis, Hydronephrosis

## Abstract

Zinner syndrome is a congenital anomaly characterized by seminal vesicle cysts, ipsilateral renal agenesis, and ejaculatory duct obstruction possibly associated with infertility. Only 200 cases of Zinner syndrome have been reported since its discovery in 1914. We present the case of a 63-year-old man seeking treatment for a ureteropelvic junction stone causing severe hydronephrosis. After the patient's history and the computed tomography findings were reviewed, the diagnosis of Zinner syndrome was confirmed. The patient has been nearly asymptomatic and has had three children during his lifetime. Our case could serve as a reference for future diagnoses of this rare anomaly.

## 1 Introduction

Zinner syndrome is an extremely rare congenital anomaly of the mesonephric or Wolffian duct. The syndrome is characterized by seminal vesicle cysts, ipsilateral renal agenesis, and ejaculatory duct obstruction. This condition was first described by Zinner in 1914, and approximately 200 cases have been reported ever since.^1^, ^2^, ^3^. The most common symptoms are urinary frequency, dysuria, and perineal pain. The enlargement of seminal vesicle cysts often leads to compression of the bladder, ureter, and reproductive system, which may cause infertility.^1^. The diagnosis often depends on radiological examination, including magnetic resonance imaging and computed tomography (CT).

In this report, we present the case of a 63-year-old man presenting with flank pain without any history of symptoms related to Zinner syndrome. However, the results of radiological examination revealed anomalies compatible with Zinner syndrome.

## 2 Case presentation

A 63-year-old man with three daughters presented to the outpatient department with left flank pain for 3 days and right flank pain for 1 day. He denied experiencing frequent urination, fever, or trauma. Physical examination revealed mild flank pain elicited by percussion. Renal ultrasound revealed a left ureteropelvic junction (UPJ) stone with severe hydronephrosis (Grade 4) in the thin cortex of the left kidney, whereas the right kidney was invisible on the ultrasound image. The patient initially hesitated when percutaneous nephrostomy was suggested. He then underwent a laboratory examination and CT scanning.

The laboratory findings indicated decreased kidney function (creatinine: 1.81 mg/dL, enhanced glomerular filtration rate: 38 mL/min/1.73 ${\;m}^{2}$) and microscopic hematuria (urine occult blood: 3+).

**Fig. 1:**
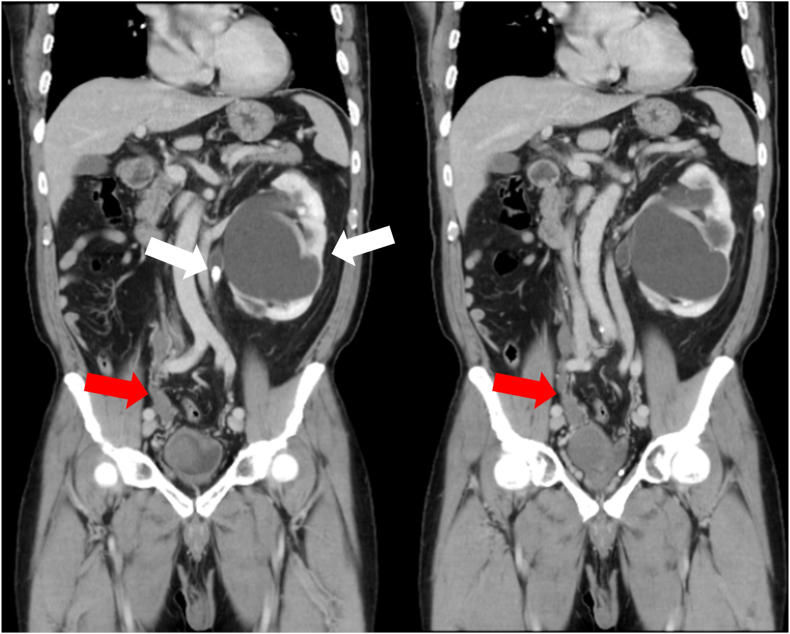
Coronal CT images revealed a left UPJ stone with moderate left hydronephrosis (white arrow) and nonvisualization of the normal right kidney. Two consecutive coronal images also revealed a dilated residual right ureter connecting to a right seminal vesicle cyst (red arrow).

**Fig. 2:**
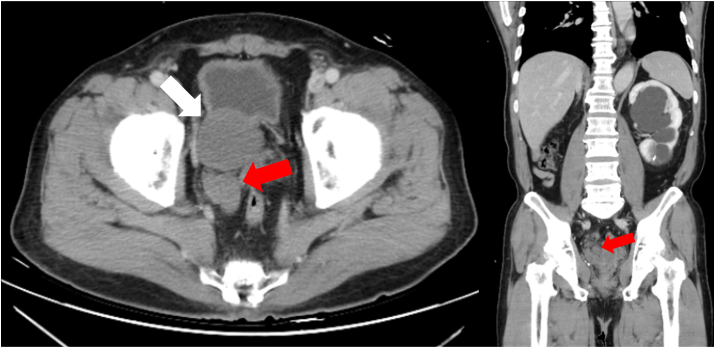
Axial and coronal CT images revealed a 48-mm right seminal vesicle cyst (white arrow) with a dilated right vas deferens (red arrow), which may indicate obstruction of the right ejaculatory duct.

A CT scan highlighted 1) a 15-mm left UPJ stone with moderate left hydronephrosis (Fig. 1); 2) a 48-mm cystic lesion in the right pelvic region (between the ureter bladder and prostate gland), indicative of a right seminal vesicle cyst (Fig. 2); and 3) nonvisualization of the right kidney with residual dilated right ureter connecting to the right seminal vesicle cyst (Fig. 1). Dilation of the right vas deferens was observed that potentially indicated an obstruction in the right ejaculatory duct (Fig. 2). Other findings from the CT scan included a double inferior vena cava and an accessory spleen.

After discussion, the patient underwent one-stage percutaneous nephrolithotripsy for the left UPJ stone. During the procedure, cystoscopy revealed that the left ureteral orifice was intact with hemitrigone, whereas the right ureteral orifice was absent with external compression from the right trigone. Left nephroscopy detected a dilated collecting system and one UPJ stone. Percutaneous nephrolithotripsy was completed with no complications. After the operation, the patient's condition was stable, and he attended regular follow-ups at our outpatient department.

Considering the CT findings of agenesis of the right kidney, residual right ureter connecting to the right seminal vesicle cyst, and obstruction of the right ejaculatory duct, we concluded that the patient has a variant of Zinner syndrome with ectopic ureteral insertion into a seminal vesicle cyst. Notable characteristics of this case are that the patient has been nearly asymptomatic and has had three children during his lifetime. Therefore, no operation or invasive treatments were planned.

## 3 Discussion

Zinner syndrome is characterized by seminal vesicle cysts, ipsilateral renal agenesis, and obstruction of the ejaculatory duct. This condition is associated with embryologic abnormalities of the mesonephric or Wolffian duct between the 4th and 13th gestational weeks. A systematic review published in 2021 reported that the mean age at diagnosis for Zinner syndrome was 29.35 years.^1^. The radiological examination findings and operation findings in the current study were compatible with the diagnosis of Zinner syndrome. However, the patient denied experiencing any common symptoms of Zinner syndrome, such as urinary frequency, dysuria, or perineal pain. A study proposed that autoantibodies against sperms could be the pathogenesis mechanism for obstruction of the ejaculatory duct in patients with Zinner syndrome.^4^. However, this etiology is not applicable for our patient, given that he has three daughters. Nevertheless, the patient with Zinner syndrome in the aforementioned study also had four biological children.^4^. The pathogenesis of infertility in Zinner syndrome remains unclear; however, evidence suggests that single-side ejaculatory duct obstruction in patients with Zinner syndrome does not necessarily lead to infertility.

In addition to the triad of Zinner syndrome symptoms, our patient had an accessory spleen and double inferior vena cava, identified in CT scans. These two symptoms can be observed in another congenital abnormality called polysplenia syndrome. In a healthy human body, the organs of the thorax and abdomen are typically asymmetric and lateralized; this arrangement is known as situs solitus. Defects in the laterality of organs can be classified into two categories: situs inversus totalis and situs ambiguous. Situs ambiguous, also known as heterotaxy syndrome, can be further classified into two major syndromes: polysplenia syndrome and asplenia syndrome. Patients with polysplenia syndrome have two or more spleens along with other anomalies of the thoracic and abdominal organs.^5^^,^^6^.

The diagnosis of polysplenia syndrome was rejected in our case because of the absence of other anomalies of the thoracic and abdominal organs. Polysplenia syndrome can be associated with left isomerism, another classification subset of heterotaxy syndrome.^7^. Patients with left isomerism often have multiple spleens, bilaterally bilobed lungs, and two morphologically left atria.^7^. Other anomalies include intestinal malrotation, preduodenal portal vein, pancreatic anomalies, and cardiac anomalies. More than 75 % of patients with polysplenia syndrome die before the age of 5 years, and only 5%–10 % of the patients without cardiac anomalies are expected to survive into adulthood without complications.^5^^,^^6^.

After the patient's history and radiological findings were reviewed, the diagnosis of Zinner syndrome was confirmed. The patient was scheduled for regular follow-up visits, considering that the solitary kidney associated with hydronephrosis may cause renal failure. Our patient had a less symptomatic UPJ stone with severe hydronephrosis and decreased renal function. Our findings indicate that patients with Zinner syndrome should be closely monitored after a diagnosis has been made.

## 4 Conclusions

Zinner syndrome is an extremely rare congenital abnormality. Our case is an example of a niche condition within Zinner syndrome and could serve as a reference for diagnosing such rare anomalies in the future. A solitary kidney may be vulnerable to renal failure if associated with kidney stone. Therefore, regular follow-ups are a crucial aspect of patient management for patients with Zinner syndrome.

## CRediT authorship contribution statement

**Tzu-Yu Chiu:** Conceptualization, Data curation, Formal analysis, Methodology, Writing – original draft, Writing – review & editing. **Szu-Ju Chen:** Investigation, Writing – original draft, Writing – review & editing. **Chun-Lin Huang:** Validation, Visualization, Writing – review & editing. **Chi-Ping Huang:** Supervision, Validation, Writing – review & editing. **Wen-Chi Chen:** Conceptualization, Data curation, Formal analysis, Investigation, Methodology, Resources, Supervision, Validation, Visualization, Writing – original draft, Writing – review & editing.

## Disclosures

The authors have no conflicts of interest to declare.
